# Comparative Assessment of the Mechanical Response to Different Screw Dimensions in Scaphoid Fracture Fixation

**DOI:** 10.3390/bioengineering12080790

**Published:** 2025-07-22

**Authors:** Esin Rothenfluh, Sambhav Jain, William R. Taylor, Seyyed Hamed Hosseini Nasab

**Affiliations:** 1 Department of Plastic and Hand Surgery, Inselspital, Bern University Hospital, 3010 Bern, Switzerland; sambhavjain394@gmail.com; 2 Laboratory for Movement Biomechanics, Department of Health Sciences and Technology, ETH Zürich, 8092 Zürich, Switzerland; bt@ethz.ch (W.R.T.); seyyed.hosseini@hest.ethz.ch (S.H.H.N.)

**Keywords:** scaphoid biomechanics, cannulated compression screw, interfragmentary displacement, scaphoid fracture fixation, bone–implant interface modelling, heterogeneous material mapping, experimental/computational modelling

## Abstract

The scaphoid is the most commonly fractured carpal bone. Headless compression screws became the gold standard for fixation, but the ideal screw diameter remains debated. This study investigates the relative benefit of using a larger screw diameter to improve stability in typical scaphoid fractures. It also examines the effects of preload and screw length on mechanical behaviour. A finite element (FE) model of a mid-waist scaphoid fracture was created. Screws from Medartis (1.7 mm, 2.2 mm, and 3.0 mm diameter; 23 mm length) were placed along the longitudinal axis. Boundary and loading conditions matched prior studies. Interfragmentary displacement (IFD) and von Mises stress were compared across screw sizes. The effects of screw length and preload were also evaluated. Maximum in-plane IFD was 2.08 mm (1.7 mm screw), 0.53 mm (2.2 mm), and 0.27 mm (3.0 mm). The 1.7 mm screw exceeded the scaphoid’s average ultimate stress (60.51 MPa). Increasing preload reduced IFD, especially above 60 N. Screws longer than 1.5 times the mid-waist diameter offered no added benefit. Larger screws provide better biomechanical fracture stability. However, the gain from 2.2 mm to 3.0 mm is minor, while 1.7 mm screws lack sufficient strength. The 2.2 mm screw offers a good balance of stability and bone preservation, making it the preferred choice.

## 1. Introduction

The scaphoid bone, which bears most of the force transmission between the hand and forearm, is the most commonly fractured carpal bone, accounting for more than 50% of all carpal bone fractures. The incidence of scaphoid fractures is second only to distal radius fractures among all wrist injuries [1,2]. They predominantly affect young individuals in their most productive working years [3]. Therefore, achieving rapid bone healing is critical for restoring wrist function during their daily activities. For this purpose, single screw osteosynthesis has become the gold standard for scaphoid fracture fixation [4,5,6,7], promoting early functional recovery given their mechanical advantages. However, despite advancements in screw designs and surgical technique, approximately 10% of fractures still fail to heal, likely due to inadequate fixation rigidity [4,8] and a tenuous blood supply in the proximal pole. Interfragmentary micromotions largely determine the healing outcome after fracture fixation [9] and should be minimized to prevent disruption of new tissue and blood vessel formation, thereby promoting the primary healing process [10].

Biomechanical studies demonstrated that centrally placed screws, in a position perpendicular to the fracture plane, provide the optimal stability of scaphoid fractures [11,12]. With regards to the choice of screw, however, there is no agreement yet on which minimum screw diameter achieves the best stability with least osseous disruption. Furthermore, the mechanical benefit in using a longer screw with different diameters has not been quantitatively assessed before. A previous cadaveric study found that fixation with longer length compression screws decreases fragment motion, possibly due to the increase in purchase [13]. From a technical point of view, longer screws may entail the risk of screw protrusion beyond the bone, which is not always easy to rule out on two dimensional X-rays during surgery. It is therefore essential to understand the relative advantage of maximizing screw length and diameter in mid-waist scaphoid fracture fixation, with respect to reducing interfragmentary motion for optimal fracture healing. Another factor contributing to the stability of the fixation is the amount of torque applied to the screws, which generates compression between both fragments. Applying sufficient torque is currently based on the surgeons’ “feeling”, but the optimum torque generated by the preload of the screw is unknown. At present there is no reference preload range with a maximum value for a safe fixation and a minimum value for sufficient compression and stability [14].

Mechanical tests of screw performance in cadaveric models, as in the aforementioned literature, provide only limited insights into real-world outcomes, while numerical simulations offer a feasible alternative that can account for the complex geometry of the scaphoid bone, the material properties, the loading conditions, and the many possibilities of incorporating fractures and implants. The aim of this study was to compare fixation efficacy of different screw diameters and lengths by assessing interfragmentary displacement and maximum stress in the mid-waist fracture planes of the scaphoid by finite element (FE) analysis.

Therefore, in this study we asked: (1) What is the relative benefit in using a larger screw diameter for fixation of the most common mid-waist scaphoid fractures, with regards to biomechanical stability? (2) Does the length of the screw influence biomechanical stability when positioned along the longitudinal axis of the scaphoid, with the screw’s midplane coinciding with the fracture plane? (3) What is the reference preload range for the optimal screw fixation of scaphoid fractures?

## 2. Methods

A finite element (FE) model of the scaphoid was previously established by the authors, based on a high-resolution computed tomography (CT) scan of a cadaveric forearm [15] with normal bony architecture of the wrist and absent osteoarthritic changes or implants. It was chosen from among four alternative cadaveric wrist samples.

### 2.1. Geometry

**Figure 1:**
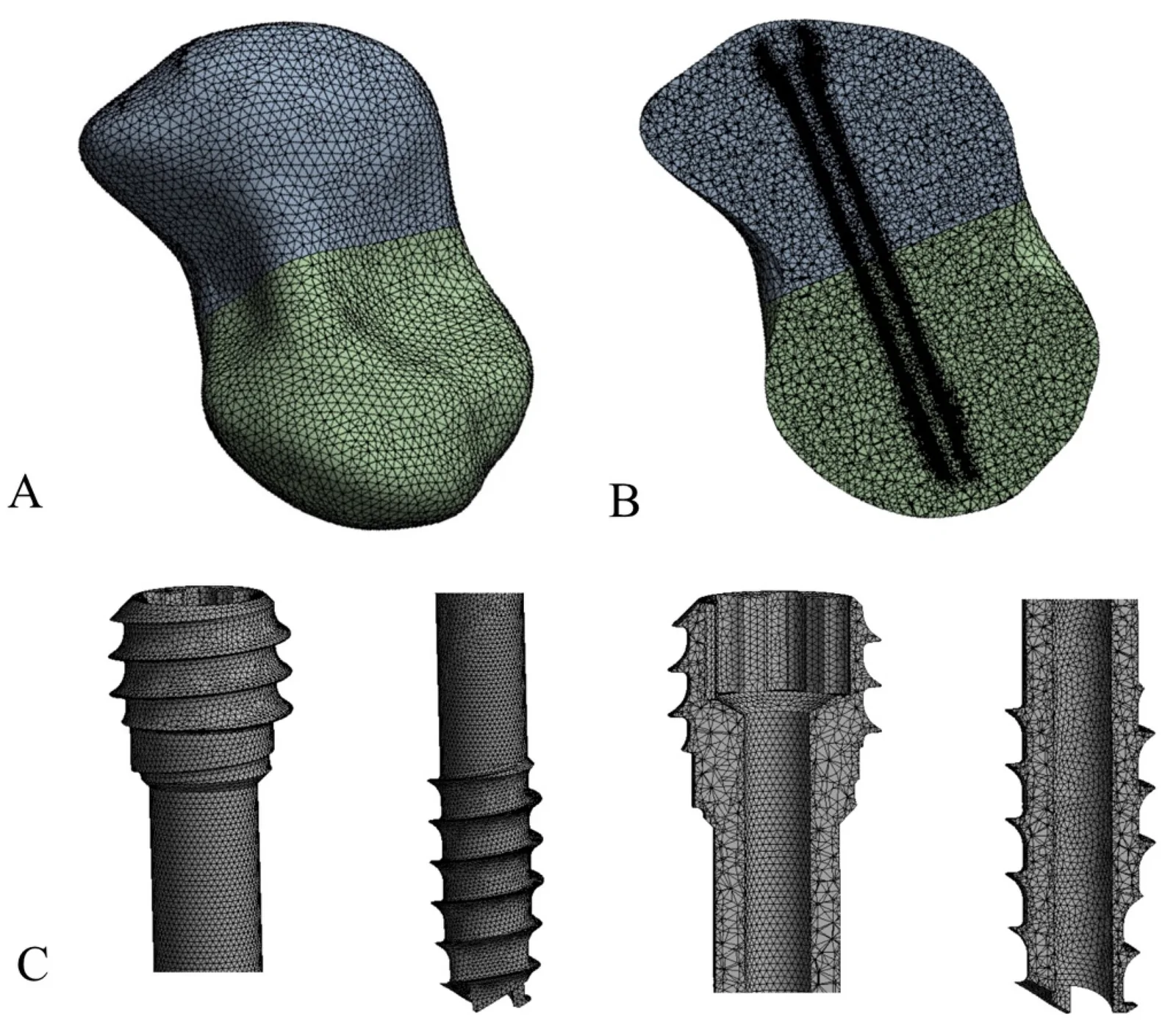
(**A**) Mesh of the scaphoid bone with a virtual fracture in the mid-waist. (**B**) Mesh in a sagittal cross section view of the scaphoid with the screw. (**C**) Detailed mesh of the screw.

The geometry of the scaphoid was extracted from the CT data and the segmentation of the scaphoid bone performed with the publicly available MITK-GEM software (Medical Interaction Toolkit Workbench [12], Version 2016.2, https://simtk.org/projects/mitk-gem, accessed on 14 July 2025). A 3D volumetric mesh was generated using tetrahedral elements and material mapping, according to the previously published description [15]. The Hounsfield unit of each voxel was used to calculate bone density (ρ_app_ in gHA/cm^3^), based on an empirical relationship, leading to the mean elastic modulus E (Young’s modulus in Megapascal, MPa) for each element according to the formula E = 6.850(ρ_app_)^1.49^ [16]. As the most frequent type of scaphoid fracture [17], a mid-waist fracture was simulated by virtual osteotomy. Specifically, the scaphoid bone was segmented into two parts, proximal and distal fragments, at the bone’s minimum cross-sectional area along the longitudinal axis. Cannulated compression screws (CCSs) by Medartis (Basel, Switzerland) with a partial thread in the common sizes (1.7 mm A-5281, 2.2 mm A-5780, and 3.0 mm A-5880) and 23 mm length were imported as CAD (computer aided design) models and positioned along the longitudinal axis, with the midplane coinciding with the fracture plane and the screw head countersunk in the subchondral distal pole. Positioning a screw along the central axis and perpendicular to the fracture plane has been shown to provide optimal stability when using a single screw [11]. For the screw length variation, 20 mm, 22 mm, and 23 mm were selected, with 23 mm representing the maximum possible length that avoids screw penetration. The body of each cannulated compression screw was subtracted from the scaphoid bone solids and the compartment of the screws, as well as both scaphoid bone fragments, were meshed with second order tetrahedral elements. A bone volume mesh size of 0.5 mm was used, which was determined through a mesh convergence study as described below. To accurately capture the screw’s geometry, finer mesh sizes of 0.2 mm and 0.1 mm were applied to the screw volume and bone-screw interface, respectively (Figure 1). Depending on the screw length and diameter configuration, the finite element mesh consisted of approximately 600,000 nodes and 400,000 elements. The screws were modelled as homogeneous, isotropic, linear elastic material with a Young’s modulus of 113.45 GPa and a Poisson’s ratio of 0.34, as provided by the company and corresponding to titanium (specification ASM136).

### 2.2. Validation

The scaphoid model was validated by the authors in an earlier study, which demonstrated that 90% of the material mapping and elastic modulus values within the mesh fell within the ranges reported in the literature [15]. The bone mesh, together with the material property data, were imported into ANSYS (Ansys^®^ Workbench, Release 2024 R1, ANSYS, Inc.; Canonsburg, PA 15317, USA.) for a sensitivity analysis. A mesh convergence study was conducted, in which the element size of bone volume was perturbed between 0.25 mm and 0.6 mm, until the variation in stress and deformation values did not significantly differ within the area of interest: the fracture plane of the upper and lower fragment. Convergence was achieved with an element size of 0.5 mm (Appendix A).

A test simulation was performed with an intact scaphoid and loading conditions copied from previous publications, showing comparable results and a realistic stress distribution and displacement [11,18].

### 2.3. Loading and Boundary Conditions

Boundary and loading conditions were applied in accordance with prior studies [15,19,20]: An area of fixed support between the proximal pole of the scaphoid and the fossa scaphoidea of the radius was defined with an exact calculation of size and location of that area with previously published data from pressure film analysis in the carpus of cadavers during load transfer experiments [20]. Considering 800 N.mm as the clinically relevant bending moment, without any additional loading through lifting weights [19,21,22], a cantilever load vector was applied to the distal aspect of the scaphoid, directed in axes of the second and third metacarpal bones [15]. With a moment arm, measured as the distance between scaphoid–radius bone interface, and for location of the cantilever load application of the presented specimen, the calculated load magnitude was 47 N. Contact was formulated using a frictional approach: a friction coefficient of 0.1 was assigned to the bone-screw and 0.4 to the bone–bone interfaces [23]. A primary preload value of 20 N was used for the simulations [11] and systematically varied between 10 N to 100 N for analysis of its influence on fracture fixation stability. The simulation time was approximately 6 h.

### 2.4. Finite Element Model Analysis

**Figure 2:**
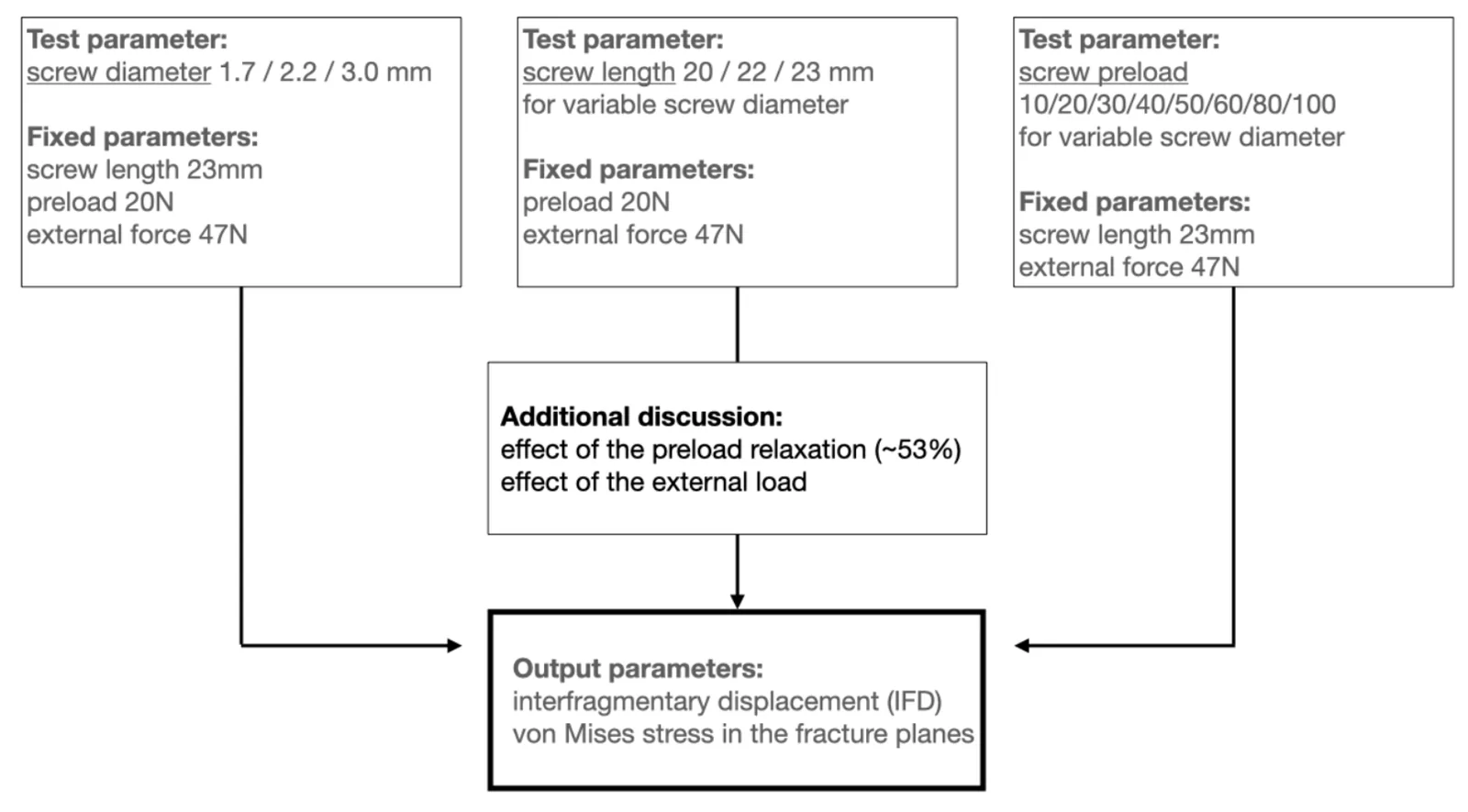
Flow chart of the simulations used to systematically investigate the influence of screw diameter, screw length, preload (with and without stress relaxation), and external loading on the stability of the scaphoid fixation.

Finite element analyses were conducted within ANSYS (Ansys^®^ Workbench, Release 2024 R1, ANSYS, Inc.). The nonlinear solution procedure used the full Newton–Raphson method, with convergence thresholds automatically controlled by ANSYS (‘Program Controlled’). The force convergence criterion was typically set to ~1% of the reference force, and the displacement convergence criterion to absolute values on the order of 10^−6^. The solver type used was the iterative the Preconditioned Conjugate Gradient (PCG) method, chosen automatically by ANSYS for this large contact model. The analytical matrix of all simulations with screw and preload variations are presented in Figure 2. While studying one parameter (screw diameter or screw length or preload), all other parameters were kept constant at their optimum. For each scenario, interfragmentary displacement IFD (mm) and maximum von Mises stress (MPa) within the fracture planes of the upper and lower fragments were compared across different screw dimensions and preload conditions. Interfragmentary displacement represents the relative motion between upper and lower bone fragments and is calculated by subtracting the nodal displacements of the lower from the upper fracture plane. As a reference for maximum von Mises stress analysis, the average ultimate strength of the scaphoid bone—calculated in a previous study using the same model (60.51 MPa)—was used as the threshold, representing the maximum sustainable stress at the onset of local material failure. For a more realistic representation of the preload, the effect of preload relaxation was taken into account and a decline of 53% was considered [24,25]. Finally, the external force was increased to 100 N to simulate the gripping of a 0.5 kg object as part of a loading configuration according to previously developed protocols [15,23].

## 3. Results

### 3.1. What Is the Relative Benefit of a Larger Screw for Stability in Mid-Waist Scaphoid Fractures?

**Figure 3:**
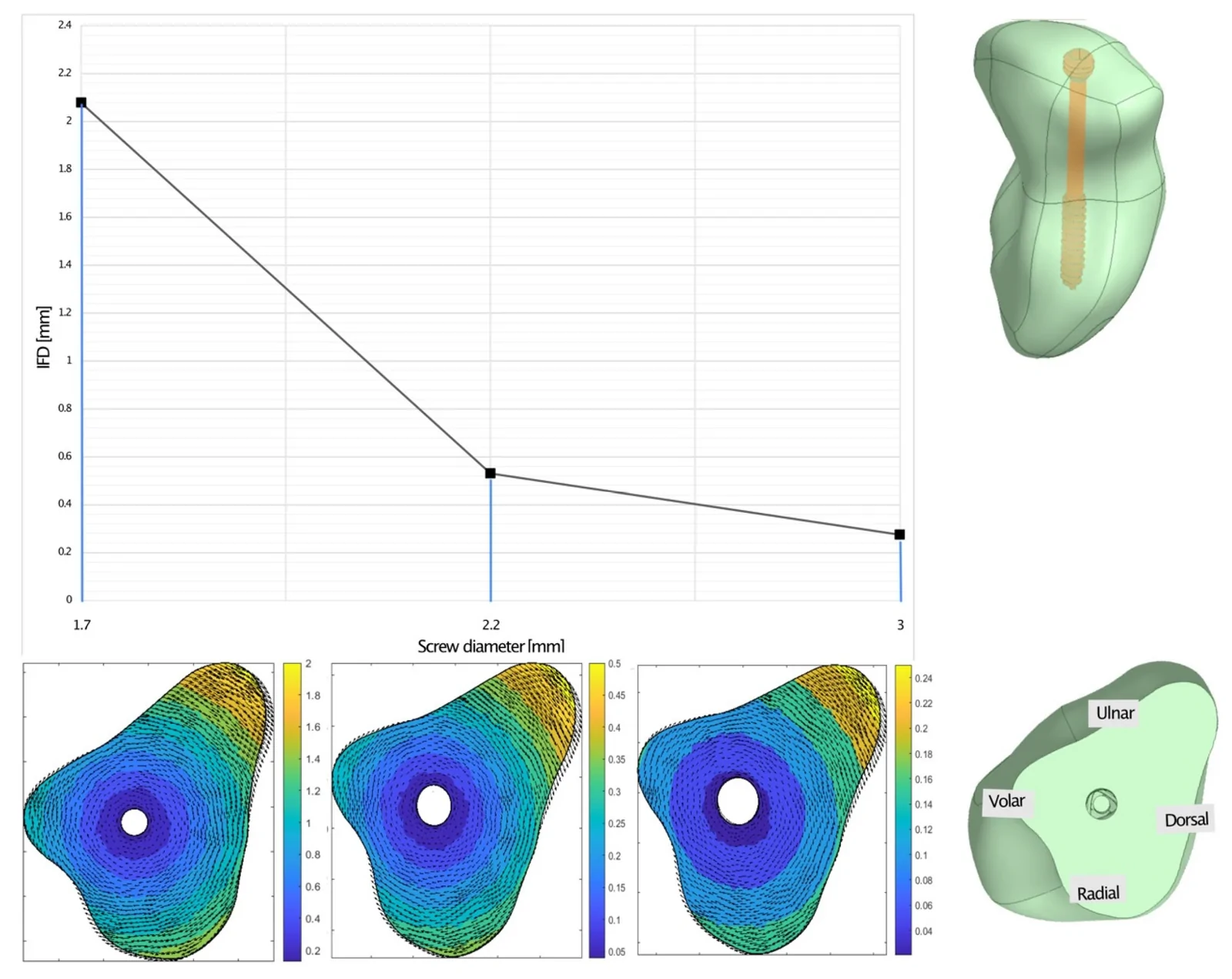
Graphic presentation of IFD against screw diameter with a cross sectional illustration of the IFD in each case. The color scale corresponds to measurements in millimeters.

**Figure 4:**
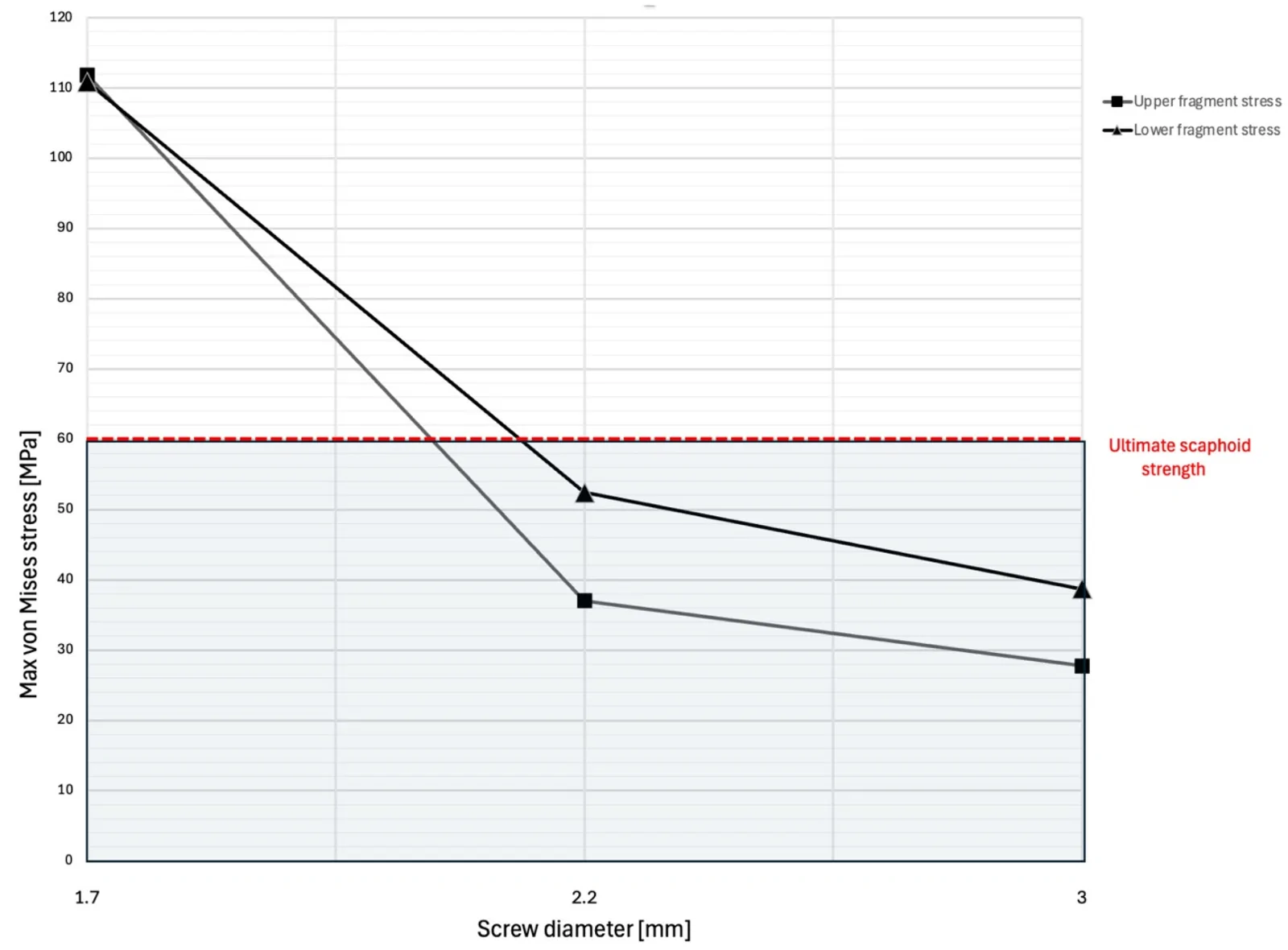
Graphic presentation of the maximum von Mises stress in the upper and lower fracture fragments against screw diameter.

The maximum IFD was 0.27 mm for the 3 mm and 0.53 mm for the 2.2 mm, while 2.08 mm was determined for the 1.7 mm screw. This resulted in a six times greater difference in the maximum interfragmentary displacement (IFD) between the 1.7 mm and 2.2 mm, compared to 2.2 mm and 3.0 mm screw diameters (Figure 3). For the 1.7 mm screw, maximum stress exceeded the average ultimate strength of the scaphoid bone (60.5 MPa): 110.9 MPa and 111.9 MPa for the lower and upper fracture planes (Figure 4). The average ultimate strength of the scaphoid bone, indicative of material failure, was not surpassed by the 2.2 mm and 3.0 mm diameter screws (Table 1).

### 3.2. Does Screw Length Affect Biomechanical Stability When Placed Along the Scaphoid’s Axis at the Fracture Plane?

**Figure 5:**
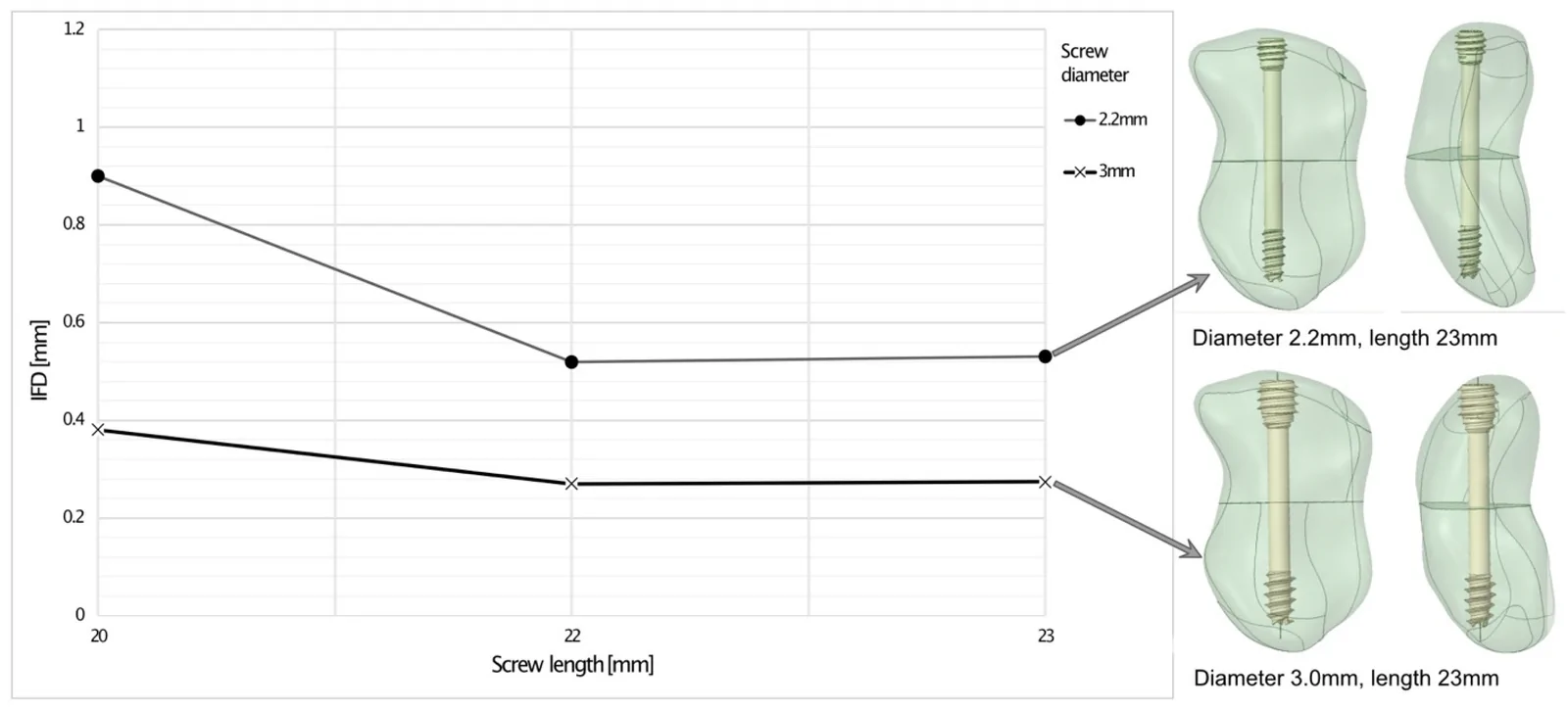
Graphic presentation of IFD against screw length for the relevant screw diameters of 2.2 and 3 mm.

According to (1), the screw diameters providing optimal stability without exceeding the material’s failure stress under the applied load were 2.2 mm and 3.0 mm. For these two screw diameters, IFD showed a slight decrease as screw length was increased from 20 to 22 mm, but remained unchanged between 22 and 23 mm. Given that the 23 mm screw approached cortical breach and that IFD values showed no significant difference between the 22 mm and 23 mm lengths, a 22 mm screw was deemed sufficient. This corresponds to 1.5× the diameter of the mid-waist fracture plane as measured on a 2D sagittal radiograph (Figure 5). Maximum stress did not exceed average ultimate strength in the upper or lower fragments for both screw lengths (22 mm and 23 mm) and diameters (2.2 and 3.0 mm) (Table 2).

### 3.3. What Is the Reference Preload Range for Fixing Scaphoid Fractures?

**Figure 6:**
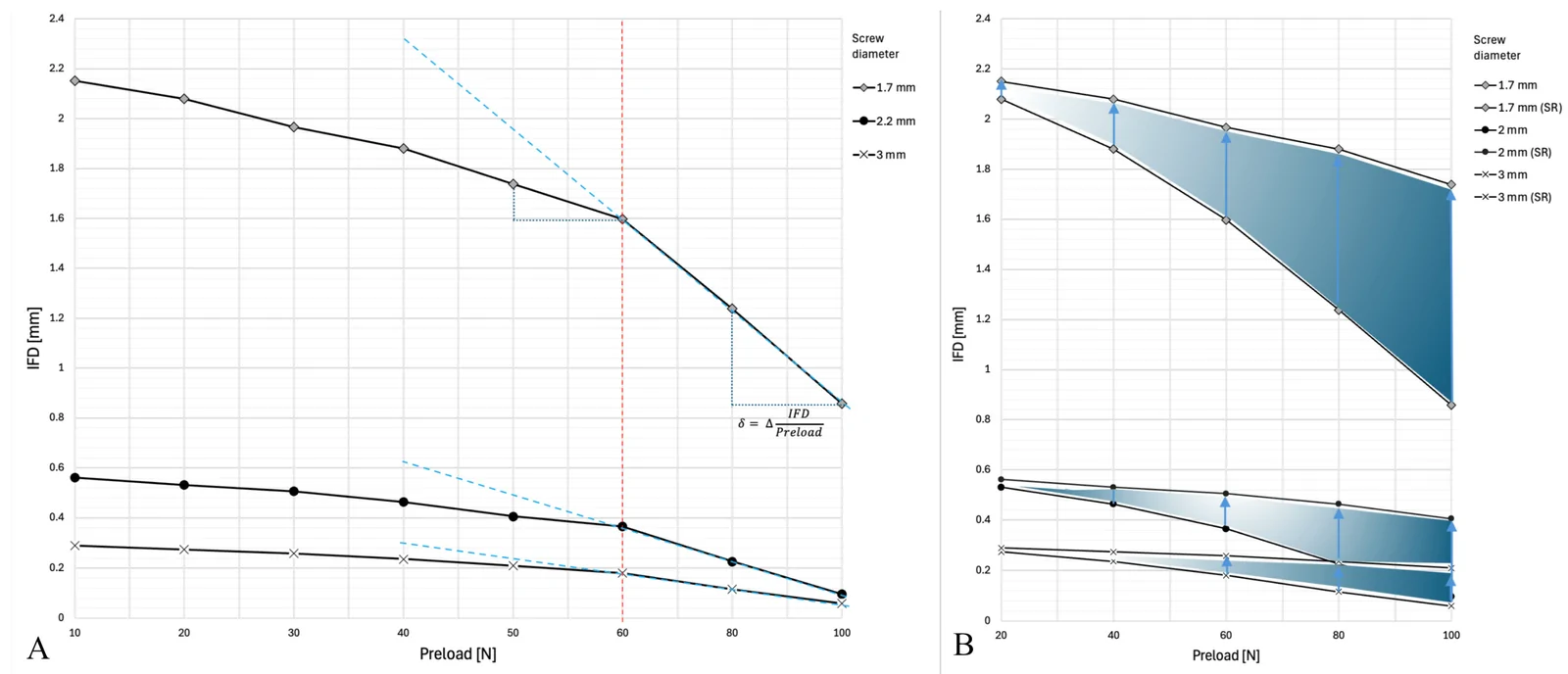
(**A**) IFD against preload for each screw diameter. The blue dashed line represents the changed slope of the curve after 60 N. (**B**) Graphic illustration of IFD against preload with stress relaxation (SR). The coloured areas between the paired curves for each screw diameter indicate the amount of difference. The arrows highlight these differences at each measured preload.

The analysis of preload variation in the screws demonstrated that increasing the preload reduces the IFD across all screw dimensions (Figure 6). Beyond a preload of 60 N, the slope increases by a factor of 2 for the 3 mm screw and by a factor of 3 for the 2.2 mm and 1.7 mm screws, indicating that 60 N may represent a threshold for achieving optimal biomechanical stability. Even at preloads up to 100 N, the 1.7 mm screw fixation, however, could not achieve an IFD comparable to that of the 2.2 mm or 3.0 mm screws. Additionally, maximum stress values exceeded the ultimate strength of the scaphoid (111.48 MPa in the upper and 98.75 MPa in the lower fragment) indicating that the 1.7 mm screw provides insufficient fixation and poses a risk of intraosseous fragmentation due to bone material failure. For the 2.2 and 3.0 mm screw diameters, maximum stress values always remained below the ultimate strength of the scaphoid in all simulations of the preload variations (Table 3).

Stress relaxation (SR) has a greater impact on screws with smaller diameters, leading to a larger increase in IFD after relaxation occurs. This is illustrated in Figure 6 by the shaded area between the paired curves (IFD with and without stress relaxation for each screw diameter). For the larger screw diameters, stress relaxation has less influence on the resultant IFD (Figure 6).

To evaluate fixation stability under varying external loads, the applied force was increased from 10 N to 47 N and 100 N, leading to a linear rise in interfragmentary displacement (IFD) and maximum von Mises stress within the fracture plane of both the upper and lower fragments across all screw diameters. For all screw diameters, maximum von Mises stress exceeded the ultimate strength of the scaphoid under an external load of 100 N, indicating that even with a 3 mm screw, the scaphoid bone would not withstand the load transmitted while holding a 0.5 kg object.

## 4. Discussion

The purpose of this study was to compare efficacy of different screw dimensions in restricting in-plane interfragmentary displacement (IFD) in a finite element model simulating the most commonly occurring mid-waist scaphoid fracture, under boundary conditions designed to replicate physiological loading. Since larger screws, both in length and diameter, are intuitively expected to offer greater mechanical stability, they may also cause more extensive disruption to the intraosseous architecture and vascularity at the microscopic level. Therefore, their relative advantage requires systematic evaluation. While this biomechanical FE analysis confirms that maximum stress and IFD within the scaphoid can be reduced by selecting a greater screw diameter for fixation of scaphoid waist fractures, it also shows that the mechanical benefit of choosing a 3 mm over a 2.2 mm screw is much less compared to a 2.2 mm over a 1.7 mm. A smaller screw is principally less invasive and allows osseous and microvascular integrity to be maintained, but fixation by a solitary 1.7 mm screw does not seem to be sufficient; hence, a 2.2 mm screw should be preferentially selected.

In selecting the appropriate screw length, our findings indicate that longer screws enhance fixation stability but simultaneously elevate the risk of cortical bone penetration. Since no difference in interfragmentary displacement (IFD) was observed between the 22 mm and the 23 mm screws, with the latter only countersunk within the distal pole by lying just beneath the surface, slightly shorter screws are preferable in this context. Nonetheless, to ensure biomechanical stability, the recommended screw length should maintain a ratio of 1.5 times the diameter of the fracture plane, corresponding to the 22 mm long screw. This ratio is dimensionally appropriate when considering the typical diameter-to-length ratio of the scaphoid waist, which is an anatomical parameter commonly referenced in orthopaedic and radiological assessments [10,15]. It is particularly useful in preoperative planning, especially when a defect in this region necessitates bone graft interposition. While the exact ratio may vary slightly depending on imaging technique and measurement plane, it typically ranges from 0.4 to 0.5 [26,27]. Consequently, a screw with a length of 1.5 to 2 times the mid-waist fracture diameter can be countersunk, but based on our findings, the factor 1.5 offers reliable biomechanical stability and reduces the risk of cortical penetration. In our analysis of the impact of screw diameter on IFD, we still used a 23 mm screw as the longest possible option, while excluding any bone penetration in the 3D model. However, this scenario can be challenging to replicate during surgery, where the cannulated screw is often inserted percutaneously. Surgeons aim to select the longest possible screw, but the intraoperative X-ray provides only two-dimensional images, making it sometimes difficult to rule out with certainty that the screw is not protruding beyond the cortical surface. Presenting a ratio is intended to provide a guide for preoperative planning of the screw length to ensure optimal stability and safe placement.

Increasing the preload beyond 60 N likely enhances stabilization by promoting improved interdigitation of the fracture fragments, an effect that is particularly evident during the initial phase of fixation, prior to the occurrence of stress relaxation. Manufacturing companies usually only evaluate the maximum compression force, under which the screw material starts to fail. The optimal torque, however, in view of limiting IFD without exceeding the ultimate strength of the bone or screw, is most often unknown and difficult to evaluate experimentally or infer from clinical studies. Estimating preload magnitude during surgical fixation remains a challenge, and the application of the appropriate torque is largely based on the surgeon’s judgment. This is particularly critical when using a cannulated compression screw (CCS), as the scaphoid bone may fail once its ultimate strength is exceeded. If this threshold is not recognized in time, the screw may breach the proximal pole. Given the current lack of clinical methods to measure or control screw preload, further investigation is warranted. Experimental studies incorporating torque meters could offer valuable insights, particularly into a surgeon’s consistency in achieving the same torque values across different time points. The future integration of torque-limiting mechanisms into these screws would represent a significant advancement in this fixation technology. Until then, however, the application of preload will remain largely dependent on the surgeon’s experience.

Previous experimental studies have investigated the impact of screw design, screw positioning in relation to the fracture plane, and the number of screws on the stability of scaphoid fractures [12,13,21,22,28]. However, all these studies were conducted on cadaveric scaphoid bones and did not examine the effect of screw dimension on fracture stability in a numerical and systematic way. Other experiments relied only on synthetic scaphoid analogues and did not account for the biological variability and local differences in bone material properties as incorporated in the present model [29,30]. In general, experimental setups inherently suffer from methodological limitations compared to finite element modelling and rarely achieve the same level of standardization. In the presented model, all boundary conditions were defined and held constant, ensuring they had no influence on the results; only the variable under investigation was systematically modified.

In addition, interfragmentary displacement (IFD) provides a more appropriate criterion for assessing fixation stability when comparing the performance of different implants, [23] as opposed to pullout force, which has been used as the outcome parameter in other studies [30]. Moreover, the effect of preloading has not been systematically assessed before in context of scaphoid fixation. It is widely recognized that the mechanical response of trabecular bone is time-dependent, yet it is often ignored for the sake of simplicity. In our analysis, we therefore accounted for stress relaxation.

The few published finite element (FE) models related to scaphoid implants have analysed the biomechanical behaviour with respect to screw positioning, using either a virtual screw [11] or a model with isotropic, homogenous material properties [18]. Other studies have focused solely on different wire configurations or the development of novel screw designs. To achieve a more realistic simulation, we incorporated the original geometry of cannulated compressions screws (CCSs), which are commonly used in hand surgical practise. A summary of the relevant implant-related, experimental, and FE studies on scaphoid fractures is provided in Table 4.

There are several limitations of this study. A single specimen with average-range anatomy was used for the FE model, but since scaphoid size and shape can vary between individuals, the results may differ with other wrist geometries. Nevertheless, our primary aim was not to provide absolute quantities for the measured parameters, but rather to compare IFD and stress values between the different screw dimensions. Different scaphoid shapes would mainly alter the magnitude, but not the trend of IFD and stress. We therefore believe that our conclusions remain valid and can deliver a useful orientation guide for fixation of scaphoid fractures.

As another limitation, the fracture geometry was simplified in the presented model with the most commonly occurring type in the mid-waist. This type of scaphoid fracture accounts for up to 85% of all cases [4] and we therefore think that they represent the most relevant collective for which treatment strategies have to be optimized. More oblique fracture planes would typically increase shear stress, and this could possibly have an impact on absolute values, but again, the impact on the trend of the results is supposedly low. Location, orientation, and variable geometries of the fracture would need extensive parameter analysis, if included, which would have been beyond the scope of the presented study. Another limitation is that the FE model was lacking direct experimental validation, and therefore we used findings of the previous literature and experiments for confirmation of reliable results, next to a sensitivity analysis of the mesh within the model [11,18,31]. Computational modelling is to some degree always a simplification of a biological system and therefore has its limitations, which also applies to the presented model. We did not include ligaments, as this would have increased processing time significantly. However, our validation results indicated that the biomechanical behaviour of the intact scaphoid model used in this study correlated well enough with results from other studies [15,18]. When ligaments are modelled as tensile spring elements, peak stress and interfragmentary displacement may vary slightly from bone-only predictions; because this deviation would be systematic, the comparative assessment of screw dimensions remains valid. The 47 N cantilever load chosen here reproduces the 800 N.mm bending moment characteristic for of daily tasks, while gripping a 0.5 kg object can impose a load of 100 N across the carpus [15,23]. To account for this higher load, we also performed an analysis at an external load of 100 N. In this case, the maximum von Mises stress exceeded the ultimate strength of the scaphoid for all screw diameters. Therefore, surgeons should view the results presented here for early rehabilitation. Despite these limitations, our FE analyses enabled the simulation of scenarios, where precise measurement of certain variables would be impossible to perform in an experimental setup. Systematic in situ assessment of stress distribution and interfragmentary stability, as presented here, is not feasible in a clinical or surgical setting.

## 5. Conclusions

- Based on this study, there is no need to use a screw with a diameter greater than 2.2 mm for biomechanically sufficient fixation of a scaphoid waist fracture, because this may compromise intraosseous perfusion.
- The screw length should not exceed 1.5 times the mid-waist diameter and must be countersunk.
- The ideal preload is approximately 60 N. However, it cannot currently be measured in situ and therefore depends on the surgeon’s experience.
- A scaphoid mid-waist fracture fixed with a single screw in an early healing stage cannot withstand external loads equivalent to gripping an object weighing 0.5 kg or more.

## Figures and Tables

**Table 1:** Interfragmentary displacement [IFD in mm] and maximum von Mises stress in the upper [UF in MPa] and lower [LF in MPa] fragments as a function of screw diameter. Shaded stress results indicate values exceeding the ultimate strength of the scaphoid bone.

		IFD [mm]	Max. Stress UF [MPa]	Max. Stress LF [MPa]
Screw diameter [mm]	1.7	2.0798	111.85	110.87
	2.2	0.5306	37.01	52.41
	3.0	0.2743	27.74	38.69

**Table 2:** Interfragmentary displacement [IFD in mm] and maximum von Mises stress in the upper [UF in MPa] and lower [LF in MPa] fragments as a function of screw diameter and length variation.

			IFD [mm]	Max. Stress UF [MPa]	Max. Stress LF [MPa]
Screw diameter and length [mm]	2.2	20	0.9002	31.19	31.66
		22	0.5197	36.95	51.79
		23	0.5306	37.01	52.41
	3.0	20	0.3805	28.16	49.58
		22	0.2701	27.24	37.96
		23	0.2743	27.74	38.69

**Table 3:** Interfragmentary displacement [IFD in mm] and maximum von Mises stress in the upper [UF in MPa] and lower [LF in MPa] fragments as a function of screw diameter and preload [in N] variation, with consideration of a stress relaxation of 53% for IFD (+SR). The orange shading highlights maximum stress values, that exceed the ultimate strength of the scaphoid.

		IFD [mm] Under Preload [N] Variation							
		10	20	30	40	50	60	80	100
Screw diameter [mm] without and with SR	1.7	2.1508	2.0798	1.9666	1.8803	1.7382	1.5971	1.2376	0.8581
	+SR		2.1508		2.0798		1.9666	1.8803	1.7382
	2.2	0.5613	0.5306	0.5055	0.4636	0.4053	0.3649	0.2258	0.0969
	+SR		0.5613		0.5306		0.5055	0.4636	0.4053
	3.0	0.29	0.5306	0.5055	0.4636	0.21	0.1806	0.1154	0.0591
	+SR		0.29		0.2743		0.2584	0.2357	0.21
		Max. stress UF [MPa] under preload [N] variation							
		10	20	30	40	50	60	80	100
	1.7	108.38	111.85	114.08	116.8	118.57	118.49	117.22	111.48
	2.2	36.62	37.01	38.47	40.26	40.53	41.31	39.5	31.82
	3.0	26.98	27.74	28.33	28.65	28.67	28.92	26.75	22.21
		Max. stress LF [MPa] under preload [N] variation							
		10	20	30	40	50	60	80	100
	1.7	113.34	110.87	108.72	107.1	105.9	104.56	101.95	98.75
	2.2	53.24	52.41	50.34	48.54	46.93	45.45	41.25	32.73
	3.0	40.47	38.69	37.2	35.85	34.43	33.08	28.73	21.92

**Table 4:** Summary of the implant related literature in the field of scaphoid fracture fixation, highlighting aspects that differ from the present study. Exp. = experimental study, FE = finite element study.

Previous Studies in the Field of Scaphoid Fracture Fixation				
Authors	Year	Exp.	FE	Scientific Question and Details
CC. Lin et al. [30]	2017	X		How do the dimension of headless compression screws alter pullout strength? - Tests performed with different screw types; - Simultaneous variation of screw diameter and length; - Output parameter: pullout force; - Tests on synthetic bone material.
S. Patel et al. [19]	2019	X		What impact does screw length have on the biomechanics of proximal scaphoid fracture fixation? - Only screw length was varied; - Only proximal pole fractures were analysed; - Output parameter: stiffness; - Tests on cadaver scaphoids.
D. Gruszka et al. [22]	2016	X		What is the impact of screw designs (four types) on the durability of fracture fixation? - Screw designs were altered, but not the dimensions of the same designs; - Output parameter: median angulation of bone fragments; - Tests on cadaver scaphoids.
J. Erhart et al. [21]	2016	X		Does an autorotational element to headless compression screws provide enhanced rotational stability in scaphoid fixations? - Screw designs were altered, but not dimensions of the same design; - Output parameter: number of cycles until a rotational clearance of 10°; - Tests on cadaver scaphoids.
A. Mandaleson et al. [28]	2018	X		How does biomechanical stability compare across three different types of fixations for scaphoid fracture non-unions? - Only non-unions were investigated, represented by a volar wedge osteotomy; - Three different fixations were analysed: (1) single screw, (2) two screws, and (3) plate osteosynthesis; - Outcome parameters: load to failure and load to 2-mm displacement; - Tests on cadaver scaphoids.
SD. Dodds et al. [13]	2006	X		How does screw length and augmentation of the screw with a K-wire influence biomechanical stability in scaphoid fracture fixation? - Only two versions of screw length for single screw fixation: “long” screw (scaphoid length: 4 mm) and “short” screw (scaphoid length: 8 mm); - Outcome parameter: interfragmentary displacement; - Tests on cadaver wrists.
WV. McCallister et al. [12]	2003	X		How does central or eccentric placement of the screw influence stability in scaphoid fracture fixation? - Only positioning of the screw in relation to the central scaphoid axis was varied; - Outcome parameters: stiffness, load to failure, and load at 2-mm displacement; - Tests on cadaver scaphoids.
S. Luria et al. [11]	2010		X	What is the optimal configuration of screw placement for scaphoid fracture fixation? - Only screw placement was varied (along the scaphoid axis, perpendicular to the fracture plane); - A virtual screw was used for simulation of fixation with a constant diameter of 3.5 mm; - Outcome parameters: interfragmentary displacement and strain.
F. Ezquerro et al. [31]	2007		X	How does the positioning of two wires influence biomechanical stability in scaphoid fracture fixation? - Only a two-wire configuration is simulated, but no screw; - Gap and angulation between two wires are varied and investigated (five configurations); - Outcome parameter: interfragmentary displacement.
B. Acar et al. [18]	2018		X	Does dorsal or volar screw fixation have an impact on stability of scaphoid fracture fixation? - Only one screw dimension is simulated (Acutrack 2 Mini, Acumed, Hillsboro); - Isotropic material properties; - Outcome parameters: relative fragment displacement/rotation and stress.
P. Varga et al. [23]	2016		X	Three different screw designs are proposed and evaluated with regard to their efficacy in scaphoid fracture fixation: (1) a single fully treaded screw (constant pitch), (2) a screw with an additional parallel, partially threaded component, and (3) a screw with an additional oblique (20°), interlocking component; - No simulation of a compression screw with variable pitch; - No systematic variation in screw dimensions; - Outcome parameter: interfragmentary motion.

## Data Availability

The original contributions presented in this study are included in the article. Further inquiries can be directed to the corresponding author.
